# The Effect of Some Natural Essential Oils Against Bovine Mastitis Caused by *Prototheca zopfii* Isolates In Vitro

**DOI:** 10.1007/s11046-018-0246-9

**Published:** 2018-01-27

**Authors:** Barbara Grzesiak, Barbara Kołodziej, Anna Głowacka, Henryk Krukowski

**Affiliations:** 1 Department of Environmental Biology, Medical University of Lodz, Żeligowskiego 7/9, 90-572 Lodz, Poland; 2 Department of Industrial and Medicinal Plants, University of Life Sciences in Lublin, Akademicka 15, 20-950 Lublin, Poland; 3 Department of Animal and Environmental Hygiene, University of Life Sciences in Lublin, Akademicka 13, 20-950 Lublin, Poland

**Keywords:** *Prototheca*, Mastitis, Essential oils, Inhibitory effect, Cow’s milk

## Abstract

The aim of the study was to evaluate the effect of essential oils obtained from *Thymus vulgaris* L., *Origanum vulgare* L., *Origanum majerana* L., *Mentha* × *piperita* L. and *Allium ursinum* L. against *Prototheca zopfii* strains that cause inflammation of the udder (mastitis) in cows. The study was conducted on ten strains derived from milk samples. The microdilution method was used to determine the sensitivity of *P. zopfii* strains to the studied essential oils, and the disk diffusion method was used to determine the sensitivity to antifungal chemotherapeutics. The plates were incubated for 48 h at 37 °C under aerobic conditions. All strains of algae were sensitive to the essential oils marjoram, thyme and oregano and resistant to mint and garlic oils. MIC values ranged from 0.25 to 1 μl/ml. Marjoram oil demonstrated the greatest activity, and oregano oil the weakest. Among the antifungal agents tested, 90% of strains showed sensitivity to nystatin. One of the tested strains (71/IV) was resistant to all investigated antifungal agents. The tested essential oils are known to have anti-algae activity and can be used as natural agents for prophylaxis in animals, particularly in mastitis-affected cows.

## Introduction

Although viruses, bacteria or fungi are commonly known to be pathogenic to animals and humans, the algae of the genus *Prototheca* may also present a threat. Potentially pathogenic species of this type are *Prototheca wickerhamii* and *Prototheca zopfii* [1].

*Prototheca zopfii* is mainly responsible for infection in animals, and the most common protothecosis is mastitis. The disease causes serious economic losses. Infection results in reduced production and poorer quality of milk, and the infected cows are usually slaughtered. The pathogen is transmitted through sick animals and the environment: drinking water, slurry, feces, the walls and floors in stalls, equipment and cups of milk [2]. A number of factors are known to promote infection, including age of cows, number of episodes, past inflammation and treatment method. The cows are more vulnerable in the early lactation stage [2, 3]. Treating *Prototheca mastitis* is difficult, and the applied therapy is beset by resistance, which may be caused by the presence of sporopollenin in the algal cell wall [2]. Commonly used antibacterial and antifungal agents are ineffective, despite demonstrating biological activity in vitro. Despite many attempts, the use of intramammary administration of levamisole and tetramisole has failed to demonstrate clinical efficacy [2, 4].

Due to the increasing resistance of microbial strains, including pathogenic algae, to synthetic drugs and the limited use of chemotherapeutics, increasing numbers of anti-algae are being sought in nature. Fortunately, plants are sources of many new and potentially effective substances [5] that may be employed in the treatment of protothecosis. The healing and antimicrobial properties of essential oils derived from plants have been known for centuries. Various studies have examined the effects of essential oil application against *Prototheca* strains [6–9].

The aim of the study was to evaluate the effect of essential oils obtained from *Thymus vulgaris* L., *Origanum vulgare* L., *Origanum majerana* L., *Mentha* × *piperita* L. and *Allium ursinum* L. against *P. zopfii* strains that cause inflammation of the udder (mastitis) in cows. The study used thyme, marjoram, mint [6, 8, 10, 11], oregano [6, 11, 12] and bear garlic oils [13, 14], which are believed to have antifungal and anti-algae effects. Peppermint oil is recommended for mastitis therapy. Japanese mint oil is a particularly effective remedy for inflammation due to its higher menthol content [15].

## Materials and Methods

### Isolation and Identification of *Prototheca zopfii* Strains

*Prototheca zopfii* isolates were obtained from a range of milk samples taken from cows with mastitis in the Department of Animal and Environmental Hygiene, University of Life Sciences in Lublin. *P. zopfii* strains were identified using standard microbiological methods: They were cultured in Sabouraud Dextrose Agar (bioMerieux, Poland), incubated for 48–72 h at 37 °C and then Gram-stained. Identification was performed using routine culture, macro- and microscopic morphological characterization and API 20C AUX (bioMerieux, Poland) [16–19]. Clotrimazole susceptibility testing using antibiotic disks (clotrimazole 50 μg) (Bio-Rad) allowed rapid differentiation of *P. wickerhamii* from *P. zopfii* [20].

### Testing the Susceptibility of *Prototheca zopfii* Isolates to Antifungal Chemotherapeutic Agents

The *P. zopfii* isolates were cultured in Sabouraud Dextrose Agar (bioMerieux, Poland) and incubated at 37 °C for 48 h under aerobic conditions. Algae suspensions were prepared with an optical density of 0.5 on the McFarland scale using a densitometer (Den-1B McFarland BioSan Densitometer). Antibiotic susceptibility was investigated by the disk diffusion method on Yeast Nitrogen Base Agar (Becton Dickinson and Company, USA) or on Mueller-Hinton II Agar (Graso, Poland). The following antifungal chemotherapeutic agents were used (Graso, Poland): nystatin (100 μg), amphotericin B (20 μg), clotrimazole (10 μg), miconazole (10 μg), ketoconazole (10 μg), fluconazole (25 μg), econazole (10 μg), flucytosine (1 μg). The results were verified after 24 and 48 h of incubation at 37 °C for all antimicrobials. The inhibitory zone diameters were measured and interpreted according to Clinical and Laboratory Standard Institute (CLSI) [21] and EUCAST standards [22].

### Essential Oils Used in the Experiments

#### Plant Material

The aboveground parts (stems, leaves and inflorescences) of five herbs species (*T. vulgaris* L.—thyme, *O. vulgare* ssp. *hirtum* (Link) letswaart—Greek oregano, *Origanum majorana* L.—marjoram, *Mentha* × *piperita* L.—peppermint, *A. ursinum* L.—bear garlic) from experimental cultivation of the Department of Medicinal and Industrial Plants, University of Life Sciences in Lublin, were collected for the study. Voucher specimens (number N-1SKK-01, N-1SKK-02, N-1SKK-03, N-1SKK-04, N-1SKK-05) are deposited at the Department of Medicinal and Industrial Plants, University of Life Sciences in Lublin. Plant material was collected in August 2016, then dried at 40 °C in a drying chamber and powdered.

#### Qualitative and Quantitative Analysis of EO

*Assay of the EO Content* The content of essential oil in herbal raw materials was assayed by indirect distillation with xylene for 3 h. The distillation was conducted in conformance with the Polish Pharmacopoeia VI [23].

*GC Analysis: Qualitative and Quantitative Analysis* A GC/MS. ITMS Varian 4000 GC–MS/MS (Varian, USA) equipped with a CP-8410 auto-injector and a 30 m × 0.25 mm i.d. VF-5 ms column (Varian, USA), film thickness 0.25 µm, was used; carrier gas He at a rate of 0.5 ml/min; injector and detector temperature 250 and 200 °C, respectively; split ratio 1:50; injection volume 5 µl. A temperature gradient was applied (50 °C for 1 min, then incremented by 4 °C/min to 250 °C, then held at 250 °C for 10 min); ionization energy 70 eV; mass range 40–870 Da; scan time 0.80 s. The oil obtained was collected in dark glass vessels, dried using dehydrated sodium sulfate and stored at below − 10 °C until chromatographic determination.

GC/FID. GC Varian 3800 (Varian, USA) equipped with a CP-8410 auto-injector and a 30 m × 0.25 mm DB-5 column (J&W Scientific, USA), film thickness 0.25 μm, carrier gas helium 0.5 ml/min, injector and detector FID temperatures 260 °C; split ratio 1:100; injection volume 5 μl. A temperature gradient was applied (50 °C for 1 min, then incremented by 4 °C/min to 250 °C, 250 °C for 10 min).

A qualitative analysis was performed on the MS spectra of the samples; the results were compared with those of the NIST library [24] and with data available in the literature [25, 26]. The identity of the compounds was confirmed by their retention indices, taken from the literature [25, 26] and our own data. The quantity composition of the volatile oil was determined by GC(FID), assuming that a total area percents of all the particular oil constituted 100%. All determinations were performed in triplicate and averaged.

### The Determination of the Minimal Inhibitory Concentration (MIC) of Essential Oils

The microbial activity of the studied essential oils was determined by the microdilution method in the broth according to NCCLS guidelines—The National Committee for Clinical Laboratory Standards based on document M27-A2 for yeast [27].

The tested strains of *P. zopfii* were cultured in a Sabouraud Dextrose Agar (SDA) bioMerieux and incubated for 48 h under aerobic conditions at 37 °C. From the inoculum, suspensions of algae were prepared in physiological saline (0.85%) with an optical density of 0.5 McFarland, which is approximately equal to 1 × 108 c.f.u./ml. To determine the density of the suspension, a Den-1B McFarland Densitometer (BioSan) was used.

The tests were performed using sterile 96-well microtiter plates (ProfiLab, Poland). The oils were diluted in DMSO. For each oil, concentrations of 32 to 0.0625 μl/ml were prepared in Sabouraud Dextrose Broth (Graso, Poland), with 100 μl of each concentration being mixed with the same volume of algae suspension. Two controls were prepared: a positive control without the essential oil and a negative control without the *Prototheca* suspension. Control media, containing only DMSO at the concentration used, did not inhibit the growth of strains. The minimum inhibitory concentration (MIC) of the test substance, i.e., its lowest concentration preventing the growth of the microorganism (no turbidity), was determined after 48-h incubation at 37 °C under aerobic conditions. To confirm the visually defined MIC, SDA plates were plated with 100 μl dilution from the last well without turbidity and the results were read after 48 h under aerobic conditions at 37 °C. All tests were performed in triplicate.

## Results

### Essential Oil Content

In total, 57 compounds were identified in thyme essential oil, seven compounds in bear garlic oil, 36 in peppermint oil, 34 in marjoram oil, and 40 in Greek oregano essential oil, including unidentified compounds. The dominant compounds in the case of thyme essential oil were thymol (51.96%), *p*-cymene (17.73%) and *γ*-terpinene (5.97%); peppermint essential oil contained mainly menthone and iso-menthone (57.57%) as well as menthol (15.32%). Sulfur-containing compounds (37.48%) were the principal components of bear garlic oil, while the main components of marjoram essential oil were trans-sabinene hydrate (38.1%), borneol (11.1%) and sabinene (7.6%). Greek oregano essential oil contained mainly carvacrol (77.29), *p*-cymene (8.85%0 and *γ*-terpinene (4.93%) (Table 1).

**Table 1:** Chemical composition of analyzed essential oil sample

Compounds	RI (acc. to Kovats)	Thyme EO	Bear garlic EO	Peppermint EO	Marjoram EO	Greek oregano EO
Cumene	928	–	–	–	–	0.06
Thujene <alpha->	931	0.92	–	–	0.50	0.50
Pinene <alpha->	938	0.78	–	0.56	1.30	0.42
Camphene	955	0.49	–	–	–	0.17
Sabinene	977	0.00	–	0.37	7.60	0.09
Pinene <beta->	982	0.20	–	0.66	0.30	0.81
1.3-Dithiane	982	–	20.06	–	–	–
Cumene	984	–	13.14	–	–	–
Octan-3-ol <1->	986	0.21	–	–	–	0.76
Disulfide, methyl 1-propenyl	989	–	6.65	–	–	–
Dimethyl trisulfide	999	–	4.59	–	–	–
Myrcene	992	0.97	–	0.76	–	–
Myrcene <beta->	993	–	–	–	1.60	–
Octanol<3>	999	–	–	0.16	–	0.06
Phellandrene <alpha->	1010	0.10	–	–	0.30	0.12
Carene <delta-2->	1011	0.10	–	–	–	–
Terpinene <alpha->	1019	1.07	–	–	4.10	0.93
n.i.	1123	–	19.79	–	–	–
Cymene <para->	1027	17.73	–	–	0.20	8.85
Limonene	1031	0.20	–	0.81	3.10	0.17
Phellandrene <beta->	1033	–	–	–	–	0.13
Cineole <1,8->	1035	0.54	–	3.88	0.11	0.04
Ocimene <€-beta->	1041	–	–	0.07	tr.	0.06
Terpinene <gamma->	1058	5.97	–	–	6.20	4.96
Sabinene hydrate <cis->	1072	0.93	–	–	6.30	0.22
Terpinolene	1085	0.09	–	tr.	1.30	–
Cymenene <para->	1095	–	–	–	–	0.03
Linalool	1099	1.53	–	0.22	–	0.07
Sabinene hydrate <trans->	1102	0.66	–	–	38.10	0.15
n.i.	1122	–	–	–	0.89	–
Menth-2-en-1-ol <cis-para->	1129	0.00	–	–	–	0.02
n.i.	1139	–	–	–	0.30	–
Sabinol <cis->	1142	–	–	tr.	–	0.02
Verbenol <trans->	1146	–	–	0.08	–	–
Isopulegol <neo->	1149	–	–	0.11	–	–
Camphor	1154	0.37	–	–	–	–
Menthone	1156	–	–	36.95	–	–
Menthone <iso->	1166	–	–	20.92	–	–
Hexane, 3-methoxy-	1167	–	18.35	–	–	–
Menthol <neo->	1170	–	–	0.89	–	–
Menthol	1178	–	–	15.42	–	–
Borneol	1180	1.45	–	–	–	0.62
Terpinen-4-ol	1188	0.46	–	–	11.10	0.39
Piperitol, cis->	1188	–	–	–	0.20	–
Menthol <iso->	1189	–	–	0.65	–	–
Cymen <8-ol-para->	1189	–	–	–	–	0.04
Dihydrocarvone <cis->	1190	–	–	–	0.20	–
Menthol <neoiso->	1192	–	–	0.05	–	–
Piperitol <trans->	1199	–	–	–	0.10	–
Terpineol <alpha->	1206	0.08	–	0.18	3.80	–
Terpineol <gamma->	1207	–	–	–	–	0.11
Dihydrocarvone trans	1210	–	–	–	–	0.09
Thymol, methyl ether	1236	0.69	–	–	–	–
Pulegone	1240	–	–	0.75	–	–
Carvacrol, methyl ether	1246	0.64	–	–	–	0.53
Terpineol <4-acetate)	1249	–	–	–	1.70	–
Linalyl acetate	1251	–	–	–	5.90	–
Piperitone	1257	–	–	1.46	–	–
Menthyl acetate <neo->	1287	–	–	4.18	–	–
Thymol	1303	52.96	–	–	0.20	1.00
n.i.	1305	–	–	–	0.50	–
Isopulegyl acetate <neo->	1307	–	–	0.08	–	–
Carvacrol	1310	3.28	–	–	0.10	77.29
Trisulfide, di-2-propenyl	1341	–	17.42	–	–	–
Bicycloelemene	1342	–	–	–	0.10	–
Neryl acetate	1370	–	–	–	0.10	–
Ylangene <alpha->	1375	0.00	–	–	–	–
Copaene <alpha->	1381	0.17	–	0.13	–	–
Geranyl acetate	1389	–	–	–	0.20	–
Bourbonene <beta->	1390	0.07	–	0.35	–	–
Elemene <beta->	1387	–	–	1.23	–	–
Caryophyllene <beta->	1426	–	–	–	2.90	
Caryophyllene <E->	1428	3.26	–	3.21	–	0.40
Copaene <beta->	1440	0.00	–	0.08	–	–
Farnesene <(Z)-beta->	1447	–	–	0.33	–	–
Aromadendrene	1449	0.00	–	–	–	–
Muurola-3.5-diene <cis->	1463	–	–	0.14	–	–
Humulene <alpha->	1468	0.11	–	0.20	0.20	0.05
Geranyl propanoate	1480	0.00	–	–	–	–
Muurolene <gamma->	1487	0.20	–	–	–	0.03
Ionone <E-beta->	1491	–	–	–	–	0.01
Germacrene D	1496	0.10	–	4.46	–	–
Bicyclogermacrene	1497	–	–	0.27	0.40	–
Viridiflorene	1503	–	–	–	–	0.00
Amorphene <gamma->	1506	0.11	–	–	–	–
Muurolene <alpha->	1511	0.13	–	–	–	–
Bisabolene <beta->	1520	0.11	–	–	–	0.41
Cadinene <gamma->	1526	0.32	–	–	–	0.04
Amorphene <delta->	1530	0.44	–	0.29	–	–
Calamenene <trans->	1535	0.14	–	–	–	–
Cadina-1,4-diene <trans->	1545	0.00	–	–	–	0.05
Cadinene <alpha->	1548	0.00	–	–	–	–
Calacorene <alpha->	1555	0.00	–	–	–	–
Germacren D-4-ol	1579	–	–	0.10	–	–
Spathulenol	1589	0.12	–	–	–	0.08
Caryophyllene oxide	1594	1.00	–	–	0.10	0.22
Humulene epoxide II	1624	0.00	–	–	–	–
Cubenol <1,10-di-epi->	1627	0.07	–	–	–	–
Eudesmol <10-epi-gamma->	1637	0.24	–	–	–	–
Cadinol <epi-alpha->	1656	0.41	–	–	–	–
Cubenol	1659	0.09	–	–	–	–
Cadinol <alpha->	1672	0.09	–	–	–	–
Eudesmol <7-epi-alpha->	1675	0.00	–	–	–	–
Caryophyllene <14-hydroxy-9-epi-(E)->	1688	0.00	–	–	–	–
Farnesol <Z,E->	1712	0.30	–	–	–	–
Monoterpene hydrocarbons		30.19	0	7.11	32.19	17.58
Oxygenated monoterpenes		4.18	0	81.86	61.1	1.49
Sesquiterpene hydrocarbons		5.16	0	10.79	3.6	0.98
Oxygenated sesquiterpenes		2.32	0	0	0.1	0.3
Phenols		57.57	0	0	0.6	78.82
Carbonilic compounds		0.37	18.35	0.08	0	0.01
Hydrocarbons		0	13.14	0	0	0
Others		0.21	48.72	0.16	0	0.86
n.i.		0	19.79	0	1.69	0

tr, trace; 0.00, peak area % smaller than 0.05% or 0.001 mg/ml; –, lack of; n.i., not identified; RI, retention index; EO, essential oil

Phenols and monoterpene hydrocarbons predominated in the samples of thyme and Greek oregano, while marjoram etheric oil was high in oxygenated monoterpenes and monoterpene hydrocarbons. These groups of compounds demonstrated the highest activity against the tested *P. zopfii* strains: Sesquiterpenes, hydrocarbons, sulfur and carbonilic compounds do not seem to be as effective in inhibiting the activity of *P. zopfii* isolates.

### Resistance to Antifungal Chemotherapeutic Agents of *Prototheca zopfii* Isolates

The *P. zopfii* strains isolated from cow milk samples were found to be highly resistant to most of the recommended chemotherapeutics for fungal cells (Table 2). None of the antifungal agents influenced strain 71/IV. All other *P. zopfii* strains were susceptible to nystatin. Three of the ten tested strains acted only this one antibiotic. Of these two strains, one was sensitive to nystatin and miconazole (166/IV), and the other to nystatin and ketoconazole (764/IV). Three of the chemotherapeutic agents tested were susceptible strains: 801/IV, 44/IV and 48/IV. Strain 49/IV was the least sensitive—four showed sensitivity to chemotherapeutics (nystatin, ketoconazole, miconazole and econazole). All tested strains were resistant to fluconazole and flucytosine.

**Table 2:** Susceptibility to chemotherapeutic agent of *Prototheca zopfii* strains

No.	Species	Strain number	Clinical material	Nystatin 100 µg	Ketoconazole 10 µg	Amphotericin B 20 µg	Miconazole 10 µg	Econazole 10 µg	Fluconazole 25 µg	Flucytosine 1 µg
1	*P. zopfii*	16/I	Milk cows with mastitis	S	R	R	R	R	R	R
2	*P. zopfii*	194	As above	S	R	R	R	R	R	R
3	*P. zopfii*	35/II	As above	S	R	R	R	R	R	R
4	*P. zopfii*	166/IV	As above	S	R	R	S	R	R	R
5	*P. zopfii*	801/IV	As above	S	S	S	R	R	R	R
6	*P. zopfii*	44/IV	As above	S	S	R	S	R	R	R
7	*P. zopfii*	49/IV	As above	S	S	R	S	S	R	R
8	*P. zopfii*	71/IV	As above	R	R	R	R	R	R	R
9	*P. zopfii*	48/IV	As above	S	S	R	S	R	R	R
10	*P. zopfii*	764/IV	As above	S	S	R	R	R	R	R

R, resistant strain; S, susceptible strain

### Susceptibility of *Prototheca zopfii* Isolates to Tested Essential Oils

All tested strains were sensitive to thyme, marjoram and oregano oils and insensible to peppermint and bear garlic oils. The marjoram oil was the most effective inhibitor of the tested strains of *P. zopfii*, with MIC values ranging from 0.25 to 0.5 μl/ml, while the least effective was oregano, with MIC values of 0.5 to 1 μl/ml. For thyme oil, the MIC values ranged from 0.25 to 1 μl/ml (Table 3).

**Table 3:** Minimal inhibitory concentration (MIC) (μl/ml) of tested essential oils

No.	Species	Strain number	Clinical material	Minimal inhibitory concentration MIC (μl/ml) of tested essential oils				
				Thyme	Oregano	Marjoram	Wild garlic	Mint
1	*P. zopfii*	16/I	Milk cows with mastitis	0.5	1	0.25	R	R
2	*P. zopfii*	194	As above	0.25	0.5	0.25	R	R
3	*P. zopfii*	35/II	As above	0.5	1	0.25	R	R
4	*P. zopfii*	166/IV	As above	1	0.5	0.5	R	R
5	*P. zopfii*	801/IV	As above	0.5	1	0.5	R	R
6	*P. zopfii*	44/IV	As above	0.5	1	0.25	R	R
7	*P. zopfii*	49/IV	As above	0.25	0.5	0.25	R	R
8	*P. zopfii*	71/IV	As above	1	1	0.5	R	R
9	*P. zopfii*	48/IV	As above	1	1	0.25	R	R
10	*P. zopfii*	764/IV	As above	1	0.5	0.5	R	R

R, resistant

The weakest tested oils worked on strain 71/IV (Table 3) which was resistant to all antibiotics used (Table 2). The most effective was marjoram essential oil (MIC: 0.5–1 μl/ml); thyme oil and oregano (MIC: 1 μl/ml) were less effective. MIC values were obtained for strain 48/IV (marjoram oil—MIC: 0.25 μl/ml, thyme oil and oregano—MIC: 1 μl/ml) (Table 3).

## Discussion

The results show that marjoram, thyme and oregano essential oils effectively inhibit the growth of *P. zopfii* strains. The essential oils contain many compounds that act synergistically and induce strong anti-algal effects. The active substances, including carvacrol and various polyphenols, are capable of dissolving the algal cell wall and penetrating into the cell, where they affect the cell metabolism [7, 28]: They cause damage to the cell membrane and cytoplasm, release proton propulsion and coagulate the cytoplasmic contents [29].

Key to this process is the lipophilic nature of the hydrocarbon skeleton and the hydrophilic nature of the functional groups. Of these compounds, the greatest activity is demonstrated by phenols, with activity falling in the following sequence: aldehydes > ketones > alcohols > esters/ethers > hydrocarbons. The most active phenols are thymol, carvacrol and eugenol, and these are present in various oils including thyme and oregano oil. Slightly less activity is demonstrated by mint and marjoram oil, as well as other essential oils which contain alcohols, e.g., terpinen-4-ol, α-terpineol and menthol [30].

The in vitro effectiveness of essential oils against *Prototheca* algae strains has also been confirmed by other researchers. Bouari (Cuc) et al. [7] tested the effects of oils from *Satureja hortensis* and *Abies alba* on *P. zopfii*. The strains of *P. zopfii* were derived from milk samples from mastitis cows and cow excrement. The MIC values for the essential oils (0.25–1 μl/ml) are the same as those for the marjoram, thyme and oregano oils. Fir oil is characterized by lower anti-algae activity (MIC: 1–4 μl/ml) than those tested in the present study.

Grzesiak et al. [9] tested eight essential oils: cinnamon (*Cinnamomum zeylandicum*), geranium (*Pelargonium graveolens*), clove (*Syzygium aromaticum*), thyme (*T. vulgaris*), lavender (*Lavandula angustifolia*), basil (*Ocimum basilicum*), rosemary (*Rosmarinus officinalis*) and clary sage (*Salvia sclarea*). The activity of these oils was tested against eight strains of *P. zopfii* isolated from milk samples taken from cows suffering from mastitis using the broth microdilution method. All studied oils were effective against algal strains, of which cinnamon, clove and thyme had the greatest effectiveness (MIC: 0.6–1 μl/ml). While the MIC values of thyme oil were found to range from 0.25 to 1 μl/ml in the present study, they were found to range from 0.6 to 1 μl/ml by Grzesiak et al. [9]. Although these are similar values, slight discrepancies may result from differences in the composition of the oil.

The *P. zopfii* strains showed low sensitivity to routine chemotherapeutics. All were 100% resistant to fluconazole and flucytosine and 90% resistant to clotrimazole, econazole and amphotericin B. The most sensitive were nystatin (90% of strains showed sensitivity) and ketoconazole (50% of strains were sensitive). Similar results were obtained by Wawron et al. [31] who conducted a study on 27 strains of *P. zopfii*. The strains of algae showed 100% resistance against clotrimazole, fluconazole, econazole and flucytosine and nearly 93% for miconazole. The most effective agents were nystatin (88.9% of strains), ketoconazole (51.9% of strains) and amphotericin B (48.1%). It is interesting that while only one strain was found to be susceptible to amphotericin B (801/IV) in the present study, Wawron et al. [31] report that almost half of the tested strains were susceptible. Grzesiak et al. found that while eight tested strains of *P. zopfii* were susceptible to nystatin, they were 100% resistant to amphotericin B, clotrimazole, miconazole, ketoconazole, fluconazole, econazole, fluorocytosine, amoxicillin, piperacillin and ceftazidime. Some strains were also resistant to gentamicin and tetracycline.

The increasing number of cases of human and animal protothecosis caused by *Prototheca* algae, including mastitis, and increasing resistance to routine antifungal chemotherapeutics [2, 32], has prompted intense research into new anti-algae agents by researchers and commercial companies. Numerous in vitro studies have shown that the oils of various plants demonstrate effective antimicrobial activity against pathogenic strains of the genus *Prototheca*, due to their high levels of polyphenols, catechins and isoflavones, together with various other biologically active compounds [33]. Studies carried out by Bouari (Cuc) et al. [8] *in vivo* on a mouse model show that mint oil (*Mentha piperita*) and savory oil (*S. hortensis*) are highly effective against skin symptoms caused by *P. zopfii*.

## Conclusions

The obtained results show that the studied essential oils can effectively reduce the growth of *P. zopfii* strains, including those resistant to antifungal chemotherapeutics used in the treatment of protothecosis. The essential oils are effective at very low concentrations and are safe.

## References

[1] Lass-Flörl C, Mayr A (2007). Human protothecosis. Clin Microbiol Rev.

[2] Lassa H, Malinowski E (2007). *Prototheca* spp. and protothecosis of animals (*Prototheca* spp. i prototekozy u zwierząt—in Polish). Life Vet.

[3] Tenhagen BA, Kalbe P, Klunder G, Heuwieser W, Baumgartner B (1998). Individual animal risk factors for *Prototheca* mastitis in cattle. Dtsch Tierarztl Wochenschr.

[4] Bergmann A (1993). Udder compatibility of tetramisole and levamisole hydrochloride and suggestion for the prescription of their intramammary use in cattle against *Prototheca zopfii*. Berl Munch Tierarztl Wochenschr.

[5] Buzzini P, Pieroni A (2003). Antimicrobial activity of extracts of *Clematis vitalba* towards pathogenic yeast and yeast-like microorganisms. Fitoterapia.

[6] Cuc (Bouari) C, Cătoi C, Fiţ N, Răpuntean S, Nadăş G, Bolfă P, Taulescu M, Gal A, Tăbăran F, Nagy A, Borza G, Moussa R (2010). The inhibitory effect of some natural essential oils upon *Prototheca* Algae in vitro growth. Bull UASVM Vet Med.

[7] Bouari (Cuc) MC, Fiţ N, Răpuntean S, Nadăş S, Gal A, Bolfă P, Taulescu M, Cătoi C (2011). In vitro evaluation of the antimicrobial properties of some plant essential oils against clinical isolates of *Prototheca* spp. Rom Biotechnol Lett.

[8] Bouari C, Bolfa P, Borza G, Nadăş G, Cătoi C, Fiţ N (2014). Antimicrobial activity of *Mentha piperita* and *Satureja hortensis* in a murine model of cutaneous protothecosis. J Mycol Med.

[9] Grzesiak B, Głowacka A, Krukowski H, Lisowski A, Lassa H, Sienkiewicz M (2016). The in vitro efficacy of essential oils and antifungal drugs against *Prototheca zopfii*. Mycopathologia.

[10] Hili P, Evans CS, Veness RG (1997). Antimicrobial action of essential oils: the effect of dimethylsulphoxide on the activity of cinnamon oil. Lett Appl Microbiol.

[11] Charai M, Mosaddak M, Faid M (1996). Chemical composition and antimicrobial activities of two aromatic plats: *Origanum majorana* L. and *O. compactum* Benth. J Essent Oil Res.

[12] Głowacka A, Bednarek-Gejo A, Budak A, Trojanowska D, Mianowany M (2012). Assessment of in vitro antifungal activity of preparation “fin Candimis” against *Candida* strains. Acta Mycol.

[13] Bagiu RV, Vlaicu B, Butnariu M (2012). Chemical composition and in vitro antifungal activity screening of the *Allium ursinum* L. (Liliaceae). Int J Mol Sci.

[14] Ivanova A, Mikhova B, Najdenski H, Tsvetkova I, Kostova I (2009). Chemical composition and antimicrobial activity of wild garlic *Allium ursinum* of Bulgarian Origin. Nat Prod Commun.

[15] Balarezo LAC, Quintana AGM. Essential topic oils on the prevention and treatment of mastitis. AgrovetMarket Animal Health. 2014. https://en.engormix.com/dairy-cattle/forums/essential-topic-oils-prevention-t30046/. Accessed 24 Aug 2017.

[16] Costa EO, Ribeiro AR, Melville PA, Prada MS, Carciofi AC, Watanabe ET (1996). Bovine mastitis due to algae of the genus *Prototheca* sp. Mycopathologia.

[17] Costa EO, Melville PA, Ribeiro AR, Watanabe ET, Parolari MCFF (1997). Epidemiologic study of environmental sources in a *Prototheca zopfii* outbreak of bovine mastitis. Mycopathologia.

[18] Marques S, Silva E, Carvalheira J, Thompson G (2006). Short communication: in vitro antimicrobial susceptibility of *Prototheca wickerhamii* and *Prototheca zopfii* isolated from bovine mastitis. J Dairy Sci.

[19] Padhye AA, Baker JG, D’Amato DF (1979). Rapid identification of *Prototheca* species by the API 20C system. J Clin Microbiol.

[20] Casal MJ, Gutierrez J (1983). Simple new test for rapid differentiation of *Prototheca wickerhamii* from *Prototheca zopfii*. J Clin Microbiol.

[21] Clinical and Laboratory Standard Institute (CLSI) Performance standards for antimicrobial susceptibility testing: nineteenth informational supplement. CLSI document M100-S19. Clinical and Laboratory Standards Institute, PA, USA; 2009.

[22] The European Committee on Antimicrobial Susceptibility Testing. Breakpoint tables for interpretation of MICs and zone diameters. Version 5.0; 2015.

[23] Polish Pharmacopoeia 6th ed. Polish Pharmaceutical Society, Polish Pharmacopoeia Commission, Office for Registration of Medicinal Products, Medical Devices and Biocidal Products, Warsaw, Poland; 2002.

[24] NIST/EPA/NIH. Mass Spectral Library with Search Program: Data Version: NIST08, Software Version 2.0f. National Institute of Standards and Technology; 2005.

[25] Joulain D, König WA (1998). The Atlas of spectral data of sesquiterpene hydrocarbons.

[26] Adams R (2004). Identification of essential oil compounds by gas chromatography/quadrupole mass spectroscopy.

[27] NCCLS. National Committee For Clinical Laboratory Standards, Reference method for broth dilution testing of yeasts, Approved standard M27-A2. 2nd ed. NCCLS: Wayne, PA; 2002.

[28] Baidar H, Sagdic O, Ozkan G, Karadogan T (2004). Antimirobial activity and composition of essential oils of *Origanum, Thymbra* and *Satureja* species with commercial importance in Turkey. Food Control.

[29] Sikkema J, de Bont JA, Poolman B (1995). Mechanisms of membrane toxicity of hydrocarbons. Microbiol Rev.

[30] Kalemba D (1998). Antimicrobial and antifungal properties of essential oils (Przeciwbakteryjne i przeciwgrzybowe właściwości olejków eterycznych—in Polish). Post Mikrobiol.

[31] Wawron W, Bochniarz M, Piech T, Wysocki J, Kocik M (2013). Antimicrobial susceptibility of *Prototheca zopfii* isolated from bovine mastitis. B Vet I Pulawy.

[32] Hazen KC (1995). New and emerging yeast pathogens. Clin Microbiol Rev.

[33] Romani A, Vignolini P, Galardi C (2003). Polyphenolic content in different plants part of soy cultivar grown under natural conditions. J Agric Food Chem.

